# Environmental and Nesting Variables Associated with Atlantic Leatherback Sea Turtle (*Dermochelys coriacea*) Embryonic and Hatching Success Rates in Grenada, West Indies

**DOI:** 10.3390/ani13040685

**Published:** 2023-02-16

**Authors:** Kate E. Charles, Clare E. Morrall, Jonnel J. Edwards, Kenrith D. Carter, Josephine A. Afema, Brian P. Butler, David P. Marancik

**Affiliations:** 1 Department of Pathobiology, School of Veterinary Medicine, St. George’s University, True Blue, West Indies, Grenada; 2 Ocean Spirits, Inc., West Indies, Grenada; 3 Department of Biology, Ecology, and Conservation, School of Arts and Sciences, St. George’s University, True Blue, West Indies, Grenada; 4 Dr Carter Veterinary Services, West Indies, Grenada

**Keywords:** leatherback sea turtle, nesting, hatchling, reproductive success

## Abstract

**Simple Summary:**

Leatherback sea turtle (*Dermochelys coriacea*) populations are experiencing significant declines and are at risk of global extinction. Sustaining reproductive success is vital to the recovery of populations. This study quantified nests and egg production between 2015–2019 on Levera Beach, Grenada and explored the association of select environmental variables with leatherback sea turtle embryonic development and hatching success rates. Annual variation was observed for the number of eggs per nest and embryo and hatching success rates. Nest location on the beach was found to be an influencing factor and development and hatching success rates decreased when nest depths were relatively shallow and when there was higher frequency of eggs with microbial growth and inspissated yolk. Relatively high temperatures recorded in a subset of nests suggest hatchling sex ratios may be skewed towards females and that increased temperatures are a risk to hatchling survival. Histopathology identified bacterial bronchopneumonia as a likely cause of morbidity and mortality in hatchlings. These findings help guide conservation strategies to support leatherback sea turtle hatchling survival.

**Abstract:**

Annual monitoring of leatherback sea turtle (*Dermochelys coriacea*) nesting grounds in Grenada, West Indies has identified relatively low hatch rates compared to worldwide trends. This study investigated the impact of selected environmental variables on leatherback sea turtle embryonic development and hatching success rates on Levera Beach in Grenada between 2015–2019. The mean number of nests per year and eggs per nest were 667.6 ± 361.6 and 80.7 ± 23.0 sd, respectively. Within excavated nests, 35.6% ± 22.0 sd of eggs successfully developed embryos and 30.6% ± 22.6 sd of eggs successfully hatched. The number of eggs per nest, along with embryo and hatching success rates, differed by nesting year. Embryo development success rate was associated with nest location, and both embryo development and hatching success rates were positively associated with nest depth and negatively associated with the percentage of eggs exhibiting microbial growth and with the presence of inspissated yolk. There was no embryo development or hatchling success association with month of the nesting season, distance from the high-water mark, distance from vegetation, nor maternal carapace length. The mean nest temperature was 31.7 °C ± 1.64 sd and mean temperatures during the middle third of egg incubation suggest clutches are highly skewed towards a preponderance of female hatchlings. Histopathologic findings in hatchling mortalities included severe, acute, multifocal, heterophilic bronchopneumonia with intralesional bacteria in 4/50 (8%) hatchlings. Data from this study guide conservation strategies by identifying risk factors and further avenues of research needed to support reproductive success of leatherback sea turtles in Grenada and the greater Caribbean region.

## 1. Introduction

Leatherback sea turtles (*Dermochelys coriacea*) are the most widely distributed of the seven marine turtle species, with foraging grounds in sub-polar waters and nesting grounds within tropical climates [1]. Populations are declining in many parts of the world [2]. Threats vary across regions and include incidental capture, legal and illegal harvesting of eggs and meat, coastal development, pollution, pathogens, and climate change [3,4]. Leatherback sea turtle populations have relatively low reproductive rates compared to other sea turtle species [5,6]. Global leatherback sea turtles hatching success ranging from 19.8% to 78.0% has been documented; Caribbean hatching success rates of 34.9–63.7% have been reported [7,8], with regional variation and inter-seasonal variation within the same nesting beach [9,10]. The drivers of hatching success variation and risk factors that accompany relatively low hatching rates are likely multifactorial and include environmental variables. Elucidating the factors affecting embryonic development and hatching success is crucial for effective leatherback sea turtle conservation and management [11].

Leatherback sea turtle reproductive success may be affected by the biological and physical characteristics of nesting beaches. Leatherback turtles prefer to nest in open areas devoid of vegetation above highwater marks [6] but these areas tend to be dynamic, with frequent erosion and accretion of sand [12]. Although beach sand cycles may contribute to cleansing the area of nest debris and microbial growth, sand movement can also increase the risk of water saturation, which negatively affects egg survival [12]. Conversely, sand or nests contaminated with infectious pathogens (including fungi and bacteria) may also negatively affect embryo and hatchling survival [8,13]. Surrounding vegetation can provide camouflage and shading of nests [14] but root growth can result in embryo mortality, as the roots can penetrate through the soft eggshells [15], prevent gas exchange between the embryos and the surrounding sand [12], potentially entrap and disorient hatchlings [16,17], and impede nesting attempts by female leatherback sea turtles (Charles, personal observation).

Changes in humidity and temperature within the nest during incubation influence embryonic and hatchling development [9,18]. Incubation temperatures below 25 °C or above 35 °C, the thermal tolerance range for leatherback sea turtles, can result in unsuccessful embryonic development and may result in hatchlings emerging with decreased levels of vigor and strength which can negatively impact post-hatching survival rates [9,11,19]. Incubation temperatures additionally govern sex development, known as temperature-dependent sex determination (TSD) [20]. To achieve a 1:1 sex ratio in sea turtles a pivotal temperature of 29.4 °C is needed during the middle one-third of the incubation period [21,22]. There is only a 2–3 °C range above and below the pivotal temperature, with cooler incubation temperatures producing predominantly male hatchlings, while warmer temperatures produce higher proportions of females [18]. This can have major impacts on population dynamics, as a 2015 study by Jensen et al. (2018) [23] described a 116:1 female bias in green sea turtles (*Chelonia mydas*) in the Great Barrier Reef, compared to a 6:1 female bias recorded 35 years prior. As global air temperatures have risen at a rate of 0.18 °C annually since 1981 [24], potential changes in environmental conditions at nesting sites can have consequences for present and future leatherback sea turtle populations.

The nation of Grenada hosts one of the largest leatherback sea turtle nesting populations in the Caribbean region [4]. The index nesting ground for leatherback sea turtles in Grenada is the 750 m long Levera Beach, at the most northernmost tip of the island [4]. Grenada’s hatchling data over the last nine years (2011–2019) shows that leatherback sea turtle hatching success ranges between 25–40% [17], which is below the global average of 50–55% [5,7]. Embryo development and hatching rates were recorded over five nesting seasons at Levera Beach (2015–2019), along with key environmental variables and egg characteristics to explore possible reasons for low reproductive success. Nest temperatures were monitored in a subset of nests (2019) to examine temperature trends through the nesting season and to examine possible effects on survival and sex ratios. Full term hatchling mortalities were examined histopathologically to investigate the impact of infectious and degenerative etiologies on survival. Results of these studies provide information on factors affecting reproductive success of threatened leatherback sea turtles in Grenada to guide future strategies of sea turtle conservation at Levera Beach in Grenada.

## 2. Materials and Methods

### 2.1. Nest Monitoring and Environmental Data Collection

Sampling methods were approved by St. George’s University Institutional Animal Care and Use Committee (IACUC-16017-R); permits were acquired from the Fisheries Division of the Grenada Government Ministry of Agriculture, Lands, Forestry, Fisheries, and the Environment.

**Figure 1:**
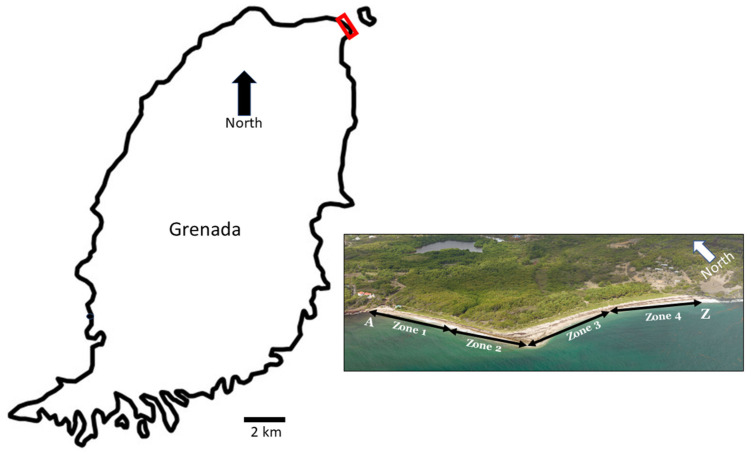
Geographic location of leatherback sea turtles (*Dermochelys coriacea*) nesting grounds on Levera Beach, Grenada (red square) and schematic of 187 m zones 1–4 and 30 m subzones A-Z used to mark and relocate nests at excavation. (Photo courtesy of Nicolas Winkler).

Data for this study were collected from Levera Beach (12°13′75″ N, 61°36′78″ W) in Grenada, from the 2015 to 2019 nesting seasons from March through August (Figure 1). The beach is within a Ramsar Protected Area and is closed to public use during the leatherback nesting season. Data collection methods are those used with nest monitoring strategies employed by Ocean Spirits Inc. [4]. Nightly beach patrols were conducted every 30 min between 19:30 and 06:00 to detect nesting female leatherback sea turtles. Biometric data, including curved carapace length (CCL) and curved carapace width (CCW), and details from Monel (National Band and Tag Company, Kentucky, USA) and Passive Integrated Transponder (PIT) tags (Biomark, Idaho, USA) were collected to associate individual turtles with nesting data.

The 750 metre white, fine sand beach was divided into four 187 metre wide zones (zone 1–4) and subdivided with wooden markers placed 30 m apart (Zones A-Z) to allow nests to be found for excavation (Figure 1). Nest distances from the seaward edge of vegetation and high-water marks were recorded. Doomed nests laid within known washout/erosion areas, below the high-water mark, or sited too close to vegetation, were relocated to an area on the beach where the nest would be at a lower risk [4]. Nest relocations were dug by hand and measured using a soft tape to ensure they were of similar depth and width to a natural nest, to ensure incubation was not impacted by the relocation. Nest relocation likely influenced environmental data and overall hatchling success recorded in this project but priority was given to efforts to support conservation efforts.

From May to September of each nesting season, 10% of the total confirmed in situ and relocated nests laid at Levera Beach were selected at random and excavated, either two days post-hatchling emergence or 70 days post-ovipositioning if emergence was not observed. This delay in excavation was to provide adequate time for complete emergence of any viable hatchlings. Nest locations were excavated to a depth of one metre deep and a width of one metre wide by hand (latex gloves were worn), the excavator determining lower sand desnity by touch. Depth to the top and bottom of the egg chamber, was determined using a soft 1 m tape.

**Figure 2:**
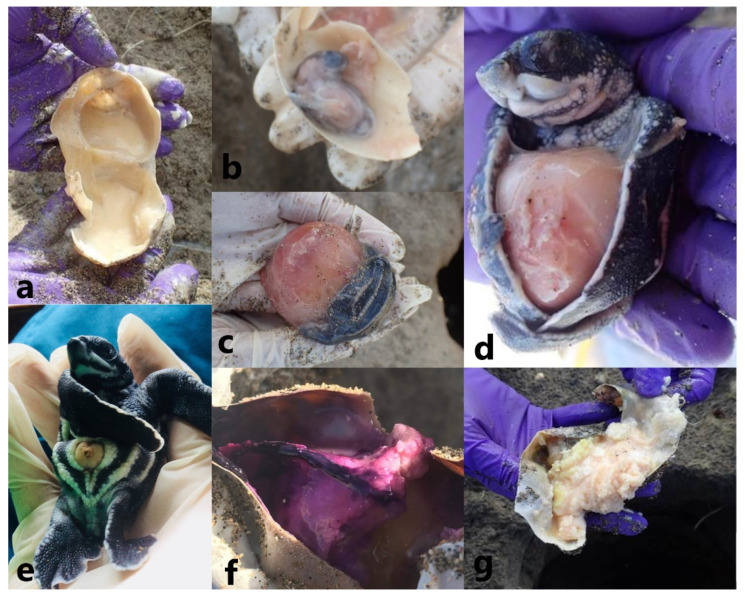
Leatherback sea turtle (*Dermochelys coriacea*) egg development stages used to characterize embryonic development success and selected observed gross changes. (**a**) No gross signs of development and variously staged embryo development at (**b**) <5 mm, (**c**) 5 mm–2 cm, and (**d**) >2 cm in size and (**e**) full term. Embryos were examined for pathologic changes consisting of yolk discolouration (**f**) and/or (**g**) yolk inspissation, (photo credit KEC).

Excavated contents were catergorised based on WIDECAST (Wider Caribbean Sea Turtle Conservation Network) protcols as being: (1) empty shells, signifying hatched eggs; (2) unhatched eggs containing embryos measuring <5 mm in length to full term; (3) yolked eggs with no gross signs of development; and (4) unfertilized shelled albumin masses (Figure 2). Embryonic development success rate was calculated as the sum of the number of hatched eggs and eggs with embryos divided by the number of laid eggs (excluding shelled albumin masses), with the embryonic development success rate representing laid eggs that had the potential to develop into hatchlings. Hatchling success rate was calculated as the number of hatched eggs divided by the number of laid eggs (excluding shelled albumin masses) [25]. This represented the percentage of eggs that hatched. Unhatched eggs were examined for the presence of visible external or internal pink to purple discolouration, interpreted to be bacterial or fungal growth, and/or inspissation of yolk which imparted a coagulated appearance to the internal contents (Figure 2).

### 2.2. Temperature Monitoring

During the 2019 nesting season, HOBO U23 Pro v2 Temperature/Relative Humidity Data Loggers (Onset, MA, USA) were placed in seven selected nests, spanning the full length of the beach during the months of March (n = 3), April (n = 2) and May (n = 2), to record data during the incubation period. Loggers were housed in 10 cm diameter polyvinyl chloride (PVC) tubes with mesh covered ends and placed mid-level within the egg chamber during ovipositioning. Loggers recorded parameters each 30 min and were removed during nest excavations. Data were uploaded into the manufacturer’s graphing and analysis software program for evaluation. Humidity data were not successfully documented by loggers. Mean nest temperatures were calculated for the total incubation period and during the middle third of the incubation period to examine potential effects on TSD. Additionally, the number of degree days above 35 °C was used as a proxy measurement of the extent to which eggs were subjected to incubation temperatures above the viable threshold for leatherback sea turtles [19]. Total degree days were calculated using the formula: total degree days = the number of degrees above 35 °C × number of days [26].

### 2.3. Histopathology

Microscopic examination was carried out on five dead late-stage embryos and/or full-term hatchlings from each of ten randomly sampled nests in 2019. Embryos were gently removed from their eggshell and any remaining egg yolk sac was discarded. The plastron was detached from the pectoral muscles with a scalpel (#10 Swann-Morton) to expose the internal organs. The internal yolk sac was removed and each embryo was placed in 10% buffered formalin for 48 h to fix the tissues. Tissues were excised from each hatchling and preserved in 70% ethanol for up to 14 days prior to being processed routinely for histopathology, embedded in paraffin, sectioned at 5 µm, and stained with hematoxylin and eosin. Based on preliminary findings and histopathologic observations of embryos from Levera Beach in previous years [8], lung, liver, skeletal muscle, and kidney were prioritized for examination.

### 2.4. Statistical Analysis

The following variables for the 2015–2019 nesting seasons were statistically examined: These included the embryo development and hatching success rates as dependent variables and distances of the nest to the high-water mark and vegetation, the depths of the top and bottom of the egg chamber, the percentage of eggs with interpreted microbial growth, the percentage of eggs with yolk inspissation, and maternal turtle CCL and CCW as independent variables. All variables except for CCL and CCW were determined to be normally distributed and normally distributed variables were examined with a one-way ANOVA to compare the means of the different numerical variables across the different months, beach locations, and years. Descriptive statistics are provided in Supplemental Files S1 and S2. If a difference was detected, then Bonferroni and Tukey’s Honest Significant Difference (HSD) post hoc tests was used to evaluate which groups were different. Results were similar between the two post hoc tests and Tukey’s HSD was utilized due to the relatively large sample size (Supplemental File S2). Data for all five years were combined into a single dataset and a simple linear regression was used to determine associations between embryo development and hatchling success rates and each continuous predictor variable (Supplemental Files S1 and S3). If a statistically significant relationship was found between an independent variable and embryo development or hatching success during simple linear regression, further analysis was performed using a multiplate linear regression (Supplemental Files S1 and S3).

A second data set was analyzed to examine nest temperature effects during the 2019 nesting season. Descriptive statistics of nest temperature and other variables recorded at each nest during the nesting season are provided. The effect of nest temperature and degree days above 35 °C on the embryo development success rate, hatching success rate, percent of eggs with microbial growth or inspissated yolk and on the depth of the top and bottom of the egg chambers was examined using simple linear regression (Supplemental Files S1 and S3). A Pearson’s correlation was run to compare degree days and the top and bottom depth of the egg chamber. Statistical analyses were performed using R Core Team (2020) [27] and GraphPad Prism 8 software (La Jolla, CA, USA). All statistics were calculated with a significance level of *p* < 0.05.

## 3. Results

### 3.1. Nesting Data and Environmental Variables

The mean number of confirmed nests per year on Levera Beach between 2015–2019 was 667.6 ± 361.6 standard deviation (sd). An annual mean of 27.7% ± 0.11 nests were manually relocated due to probable exposure to water inundation and/or vegetation growth. The mean number of eggs per nest was 80.7 ± 23.0 sd with a significantly higher number of eggs per nest in 2018 compared to other years (*p* < 0.01) (Table 1). Within excavated nests, the embryonic development rate (eggs with embryo development) was 35.6% ± 22.0 sd and the hatch rate was 30.6% ± 22.6 sd. There was significantly higher embryonic development (*p* < 0.001) and hatching rates (*p* < 0.001) in 2015 compared to other years although the environmental variables monitored in 2015 were not significantly different in this year to explain this finding (Table 1, Supplemental File S2). Neither the embryo development nor the hatching success rates were significantly associated with the month of the nesting season when all years were examined together. There was a significant association between the embryo development rate and nest location (*p* = 0.03) with a trend towards a higher percentage of embryos developed in the second and third quarters of the beach (the middle sections); however, this association was not statistically significant (*p* = 0.06) and did not translate to there being a significantly higher percentage of hatching success (*p* = 0.121) (Supplemental File S2).

Combined data from nests excavated between 2015–2019 were used to examine the effects of environmental variables and egg conditions on reproductive success (Supplemental File S3). Simple linear regression results demonstrated that embryo development success rates and hatching success rates were positively associated with the depth of the top and bottom of the egg chamber (*p* < 0.001) (Table 1, Supplemental File S3). Embryo development success rates increased by 0.50% and 0.43% for every one cm increase in the depth of the top and bottom of the egg chamber, respectively (*p* < 0.001) (Supplemental File S3). Correspondingly, the hatching success rates increased 0.50% and 0.34% for every cm increase in depth of the top and bottom of the egg chamber, respectively (*p* < 0.001) (Supplemental File S3). The depth of the top and bottom of the egg chamber was significantly lower in 2018 compared to other years (*p* < 0.001) (Table 1, Supplemental File S3) and was significantly lower in the first zone of the beach compared to other locations (*p* = 0.02) (Supplemental File S2).

Multiple linear regression analysis showed embryo development and hatching success rates were negatively associated with the presence of microbial growth and the presence of inspissated yolk (*p* < 0.001 for each variable) (Table 1, Supplemental File S3). Embryo development and hatching success rates decreased 0.57% and 0.55%, respectively, for every 1% increase in eggs with microbial growth (*p* < 0.001) (Supplemental File S3). The percentage of eggs with microbial growth significantly differed by month (*p* = 0.01) and year (*p =* 0.04) but comparisons between individual months and years were not significant when analyzed by a one-way ANOVA (Supplemental File S2). There was no significant association between nest location and the percentage of eggs within those nests with microbial growth.

Multiple linear regression analysis showed for each percentage point increase in the presence of eggs with inspissated yolk, embryo development and hatching rates decreased by 0.37% and 0.27%, respectively (*p* = 0.01) (Supplemental File S3). There was a significantly higher percentage of inspissated eggs in 2018 (*p* < 0.001) and 2019 (*p* < 0.001) compared to 2016 (Table 1, Supplemental File S2) and in the first quarter of the beach compared to the fourth quarter (*p* = 0.03) with no association with month of the nesting season (Supplemental File S2).

There was no association between embryonic development and hatching success rates and the measured high-water mark (27.8 m ± 7.7 sd), distance from vegetation (mean 12.3 m ± 5.7 sd), or CCL of the adult nesting female (153.9 cm ± 11.1 sd) (Supplemental File S2).

### 3.2. Temperature Monitoring

**Figure 3:**
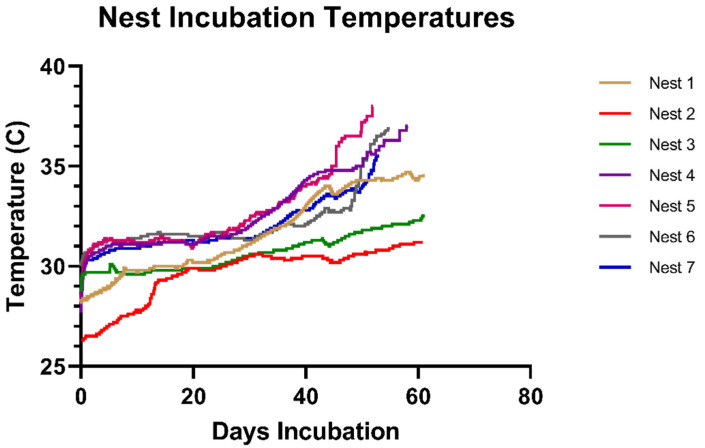
Time-series plot of nest temperatures recorded by temperature loggers deployed in leatherback sea turtle (*Dermochelys coriacea*) nests in March (Nest 1–3), April (Nest 4–5), May (Nest 6–7) in 2019 on Levera Beach, Grenada.

The mean and range of nest temperatures, and calculated degree days from seven temperature loggers recorded in 2019 is presented in Table 2 and the time series plot is presented in Figure 3. Mean temperatures in all seven nests exceeded the pivotal temperature of 29 °C needed for a 1:1 sex ratio during the middle third of the incubation period [21,22] (Table 2). Five nests demonstrated between 3.0 and 18.7 degree days above the 35 °C thermal tolerance range for leatherback sea turtles [9,18] (Table 2). The embryo development and hatch rates and the percent of eggs with microbial growth or inspissated yolk were not significantly associated with mean nest temperature or the number of degree days above 35 °C, although the small sample size hinders adequate statistical evaluation (Table 2). Mean nest temperatures decreased by 0.1 °C for every centimetre increase in depth of the top and bottom of the egg chamber (*p =* 0.05) (Table 2, Supplemental File S3). There was significant and high negative correlation between degree days above 35 °C and the top depth of the egg chamber (*p =* 0.02, r = −0.826) and bottom depth of the egg chamber (*p <* 0.0001, r = −0.966) (Table 2).

### 3.3. Histopathology

**Figure 4:**
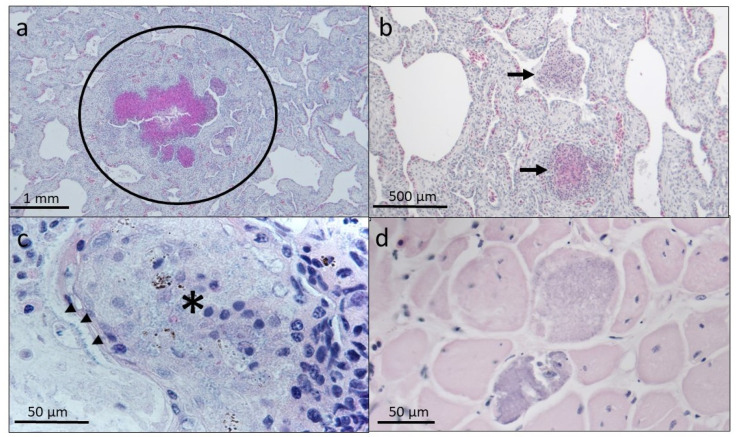
Histopathologic findings in leatherback sea turtle (*Dermochelys coriacea*) post-hatching mortalities. (**a**) Large focus of heterophilic debris and necrosis within the airway of a bronchi (circled). (**b**) Multiple areas of cellular and heterophilic debris within falveolar spaces (arrows). (**c**) Cellular and acellular debris within faveolar spaces is associated bacteria lining the bronchi epithelium (arrowheads). (**d**) Disruption of the sarcolemma of two skeletal muscle bundles by mineral deposits.

Histopathologic changes were observed in 11/50 (22%) embryos examined in 2019. The most clinically significant finding was moderate to severe, acute, multifocal, heterophilic bronchopneumonia in 4/50 (8%) hatchlings (Figure 4). Gram-negative rods were visible in 3/4 (75%) of hatchlings with bronchopneumonia in Gram-stained histopathologic slides of lesions. Three of the four hatchlings with bronchopneumonia were from the same nest and all four hatchlings were from nests laid in June 2019. Additional histopathologic changes noted included mild, multifocal, skeletal myofiber necrosis and mineralization in 7/50 (14%) hatchlings (Figure 4). All hatchlings with muscle lesions were from different nests that were laid in March, April, and May 2019.

## 4. Discussion

The objective of this study was to examine extrinsic factors that may be associated with leatherback sea turtle hatching rates on Levera Beach in Grenada. Embryonic development and hatching success were positively and negatively associated with multiple variables, but the level of each influence by itself was relatively low, suggesting influences are likely multifactorial and intertwined. Disentangling and addressing these variables can guide conservation strategies with the goal of increasing the relatively low hatch rates in Grenada [4,10] and improving reproductive success of these threatened animals. For example, artificially controlling nesting conditions by altering nest depth and providing additional shade in situ may reduce the nest temperatures, potentially increasing survival and supporting more male hatchlings to develop.

Embryonic development was significantly associated with the location of the nest on the beach, which supports previous reports that leatherback sea turtles have adapted to selecting beaches with deep water approaches and open sand, rather than primarily selecting specific ideal nesting sites on a beach [28,29]. This observation is consistent with documented failed nesting attempts at Levera Beach due to erosion or high-water tables, while adjacent, seemingly more suitable beaches with little erosion or inundation are not frequented despite being more hospitable [17]. Reproductive success was not found to be significantly associated with the distance of the nest from the high-water mark or vegetation although this has been observed in the past on Levera Beach [17] and described in previous studies [16,29]. This likely results from efforts to relocate nests from high-risk areas to prevent high water or vegetation from negatively impacting nests. This supports the practice of egg relocation to more suitable beach sections and the need for further research at Levera Beach to refine this practice. However, as environmental loggers failed to record nest humidity levels, the overall effect of subsurface moisture from tidal effects and rainfall cannot be ruled out as an influencing factor on embryonic and hatching success rates.

Nest depth was found to be an important factor in reproductive success, as hatching success rates increased by 0.3–0.45 percentage points for every 1 cm increase in the depth of the egg chamber from the surface. Amongst all the sea turtle species, leatherback sea turtles dig the deepest egg chambers, which can increase the risk of sea water inundation but also provides cooler temperatures with less fluctuations [30]. Both temperature and number of degree days >35 °C decreased with increased nest depth, although these changes alone were not enough to produce statistically significant associations of temperature or degree days with reproductive success; however, there was substantial temperature variation between individual nests, and a relatively low number of nests were monitored with temperature loggers, which likely affected statistical confidence. The positive association with nest depth and embryonic and hatching success suggests that nest relocation on Levera Beach should include burying eggs at the deeper depth range of leatherback sea turtle chambers, although optimal nest depth needs to be further examined.

Although mean nest temperature and degree days were not found to be statistically associated with embryonic or hatching success rates, the relatively high temperature recordings are of concern. Maximum nest temperatures ranged from 31.4 °C to 38.8 °C for all seven nests during the late stages of development, including up to 18 degree days above 35 °C, indicating the eggs are subjected to prolonged periods of temperatures above the stated thermal tolerance threshold. Further research is needed to better understand whether this is associated with embryo development and hatchling emergence [8,31]. The temperature readings from this study also suggest that sex ratios in hatchlings may be skewed towards female predominance. All seven nests had mean temperatures during the middle third of incubation above the 29.4 °C pivotal temperature needed to gain a 1:1 male:female ratio [22]. These temperatures ranged from 30.3–32.6 °C, which is also above the stated 29.4 °C threshold for producing 100% female offspring [30]. Further research should be prioritized in this area, including monitoring temperatures in a larger subset of nests for subsequent years and confirmation of hatchling sex. Additionally, in addition to nest relocation, additional mitigation strategies may be needed to manipulate nesting temperatures. Various factors can influence sand temperatures, including seasonal climate, sand colour, rainfall, and daily hours of shading [9]. The use of artificial shading and alteration of humidity may be beneficial in supporting reproductive success [32,33]. Development of sea turtle hatcheries have been shown to provide substantial control over environmental variables, resulting in increased hatchling survival [34,35]. This may become more important as climate change adds to reproductive pressures upon leatherback sea turtles [36]. However, the male:female ratio needed to maintain sea turtle populations is unknown at this time. This needs to be determined to support conservation of at-risk populations.

Although hatcheries can provide pivotal temperature regulation, they also are associated with a higher risk of pathogens causing mortalities in eggs and hatchlings than in situ [37]. In our study, there was evidence that reproductive success was affected by infectious disease. Embryonic development and hatchling survival was negatively associated with the percentage of eggs that had microbial growth and inspissated yolk, the latter of which has been associated with infectious disease in eggs in poultry [38,39]. However, it is unknown if these changes may represent primary, secondary, or post-mortem changes. Inspissated yolk has also been suggested to represent coagulation secondary to high temperature, although to date there is no evidence to support this theory [8]. Eight percent of the hatchlings examined histopathologically had bronchopneumonia associated with intralesional gram-negative bacterial rods. This is consistent with similar levels of bronchopneumonia found by Choi et al. (2020) in previous years on Levera Beach and may represent infection by a primary pathogen or one or more opportunistic pathogens secondary to host stress or immunosuppression [40]. Bacteria may pass through the porous eggshells and reach the developing embryos or directly infect hatchlings [41]. There is also the possibility that bacteria are transferred by the nesting female during oviposition, from her body fluids [42,43]. Several potential opportunistic bacteria have been isolated from the external surface of leatherback sea turtle eggs and in tissues from hatchling mortalities [44,45]; however, the relationship between bacteria and disease is unknown. Further pathogenicity and epidemiologic studies are warranted to identify bacteria observed in this study and to examine host, pathogen, and environmental factors that may govern these interactions.

## 5. Conclusions

This study documented multiple external factors associated with leatherback sea turtle embryonic development and hatching success rates on Levera Beach in Grenada. These findings indicate that reproductive success varies by year and by site on the beach and is positively associated with relatively deeper nest depths, which may provide more optimal incubation temperatures. Other factors such as predation were not examined but may negatively impact reproductive success and warrant examination in future studies. Further research is needed to examine temperature trends within nests during additional nesting seasons to more comprehensively describe the potential effect on hatchling survival and sex determination, as temperature readings were critically high. The presence of microbial growth and inspissated yolk were associated with decreased embryonic development and hatching success rates, and may represent infectious processes. Infectious agents, especially those observed in hatchlings with bronchopneumonia, warrants further examination to identify pathogens, and whether environmental factors may be predisposing eggs and hatchlings to infection. Current strategies to relocate nests further from the high-water mark and vegetation appear to have a positive effect, and data from this study will help guide additional techniques to support reproductive success, such as burying eggs at optimal nest depth and reducing temperatures in nests with natural or artificial shading. This baseline data can also be used to examine if these nesting variables are also influencing factors in other regions of the Caribbean. This will aid in identifying trends and help develop the best conservation strategies to ensure future population sustainability for leatherback sea turtles.

## Figures and Tables

**Table 1:** Annual leatherback sea turtle (*Dermochelys coriacea*) nesting data from 2015–2019 on Levera Beach, Grenada. Data is represented as mean ± standard deviation (sd) and significant differences between years are designated with a different superscript letter within each column.

Year	Confirmed Nests	Number of Nests Excavated	Eggs per Nest	Eggs with Embryo Present per Nest	Hatched Eggs per Nest	Eggs with Microbial Growth per Nest	Eggs with Inspissated Yolk per Nest	Depth of Top of Egg Chamber (cm)	Depth of Bottom of Egg Chamber (cm)
2015	967	94	77.6 ± 18.1 ^a^	45.5 ± 16.9 (59.7%) ^a^	35.2 ± 14.1 (47.5%) ^a^	6.3 ± 9.6 (8.1%) ^a^	NA	60.6 ± 10.0 ^a^	80.2 ± 11.3 ^a^
2016	915	99	79.9 ± 23.3 ^a^	32.6 ± 19.6 (39.8%) ^b^	23.2 ± 17.4 (30.5%) ^b^	3.6 ± 6.6 (4.2%) ^a^	0.7 ± 2.5 (0.7%) ^a^	50.6 ± 11.5 ^a^	77.0 ± 11.2 ^a^
2017	412	123	76.1 ± 24.5 ^a^	29.7 ± 20.3 (40.5%) ^b^	22.7 ± 15.2 (25.0%) ^b^	4.8 ± 7.4 (2.8%) ^a^	3.4 ± 9.3 (0.04%) ^b^	54.8 ± 14.5 ^a^	77.4 ± 14.6 ^a^
2018	609	49	90.9 ± 30.6 ^b^	31.4 ± 17.5 (36.4%) ^b^	24.1 ± 15.6 (28.8%) ^b^	3.7 ± 6.9 (4.2%) ^a^	5.1 ± 9.9 (0.1%) ^c^	46.2 ± 11.9 ^b^	68.4 ± 11.6 ^b^
2019	435	129	81.2 ± 20.9 ^a^	27.9 ± 17.1 (35.6%) ^b^	23.5 ± 16.5 (30.6%) ^b^	3.9 ± 10.4 (5.3%) ^a^	6.4 ± 10.9 (0.5%) ^c^	58.6 ± 12.3 ^a^	77.9 ± 10.4 ^a^

**Table 2:** Mean and range of nest temperatures and calculated degree days and associated variables in seven leatherback sea turtle (*Dermochelys coriacea*) nests during the 2019 nesting season at Levera Beach, Grenada. Temperature loggers were deployed for 60–80 days during nest incubation.

	Date Deployed	Mean and Min-Max Nest Temperature (°C)	Degree Days >35 °C	Mean TSD Period Nest Temp (°C)	Embryos Developed (%)	Hatched Eggs (%)	Eggs With Microbial Growth (%)	Eggs With Inspissated Yolk (%)
Nest 1	30 March	32.4, 28.3–36.1	6.3	31.4	52.3	31.8	0.0	1.0
Nest 2	30 March	30.3, 26.3–31.4	0.0	30.3	0.0	0.0	76.9	23.1
Nest 3	31 March	30.9, 28.9–32.9	0.0	30.6	51.0	46.1	2.9	26.5
Nest 4	25 April	33.4, 31.4–37.7	17.9	32.5	20.9	18.7	0.0	1.1
Nest 5	25 April	33.4, 28.4–38.8	18.7	32.6	27.5	22.5	0.0	1.3
Nest 6	21 May	32.7, 30.0–37.0	17.2	31.7	39.1	34.8	5.8	5.8
Nest 7	21 May	29.1, 27.0–35.7	3.0	31.8	49.5	49.5	0.0	1.0

TSD: Temperature-based sex differentiation.

## Data Availability

Supporting data is available upon request to the corresponding author (kate@oceanspirits.org).

## Supplementary Materials

The following supporting information can be downloaded at: https://www.mdpi.com/article/10.3390/ani13040685/s1, Supplemental File S1; Supplemental File S2; Supplemental File S3.
