# Supplementary material for: Environmental and Nesting Variables Associated with Atlantic Leatherback Sea Turtle (Dermochelys coriacea) Embryonic and Hatching Success Rates in Grenada, West Indies
Source: Animals (Basel). 2023 Feb 16;13(4):685. doi: 10.3390/ani13040685 (PMC9951857; doi:10.3390/ani13040685)
Supplement: Supplementary file 1 [file animals-13-00685-s001.zip › Supplemental File 1.pdf]

One-way ANOVA analysis

One-way ANOVA was used to compare the mean percent values of embryo development success and hatching success across different months, years and locations. Eggs were laid in the months of March, April, May and June and there were no differences in embryo development success or hatching success between months (p= 0.722 and p=0.23). One-way ANOVA revealed statistically significant differences in the percent of embryo development success and percent hatching success among the different years (supplemental table 2). Tukey HSD and Bonferroni correction showed the percent of embryo development success and percent hatching success in 2015 was significantly different (higher) compared to the years 2016, 2017, 2018 and 2019 (supplemental table 2). One way ANOVA revealed statistical difference in hatching success between the locations, and Tukey HSD showed some difference in hatching success between locations 2 and 3 (p=0.06).

Simple linear regression analysis

Simple linear regression was performed to establish the relationship between various independent environmental variables (top of egg chamber, bottom of egg chamber, microbial growth, high water mark, distance from vegetation) and hatching success or embryo development (dependent variable).

To ensure assumptions for normality were not violated, the distributions of the dependent variables (hatching success and embryo development success) were assessed using histogram plots. Assumptions for linearity were assessed by plotting each independent variable against hatching success or embryo development. Carapace length did not meet the assumptions for normality and linearity and was excluded from analysis.

The results for relationships of the environmental variables and hatching success are presented in supplemental file 3. There was a positive relationship between the depth of the top of the egg chamber and hatching success (there was a 0.5% increase in hatching success for every 1cm increase in the depth of the top of the egg chamber). Similarly, hatching success increased by 0.34% for every cm increase in the depth of the bottom of the egg chamber. There was a negative relationship between microbial growth and hatching success: hatching success decreased by 0.54% for every 1% increase in microbial growth. However, there relationship between distance of nest from vegetation or high-water mark with hatching success were not significant.

Multiple regression analysis

Distance of egg nests from vegetation and high-water mark were excluded from multiple regression analysis because the relationship with hatching success was not significant. The depth of the bottom of the egg chamber was excluded due to high correlation (0.7436) with the top of the egg chamber. There was a significant negative relationship between microbial growth and hatching success: for every 1% increase in microbial growth, hatching success

decreased by 0.69%. There was a positive relationship between the depth of the top egg chamber and hatching success: for every 1cm increase in the depth of the top of the egg chamber, hatching success increased by 0.4%. Hatching success for years 2016, 2017, 2018 and 2019 decreased by 19.8%, 23.1%, 15.0% and 18.0% respectively compared to the hatching success in 2015.

Data on eggs with inspissated yolk was not collected in 2015 and data is available from 2016 - 2019. Multiple linear regression was done to establish the effect of microbial growth, depth of top of the egg chamber and % eggs with inspissated yolk on hatching success. Hatching success decreased by 0.55% for every 1% increase in microbial growth, and hatching success decreased by 0.27% for every 1% increase in eggs with inspissated yolk.
